# Notes of the Week

**Published:** 1921-06-04

**Authors:** 


### NOTES OF THE WEEK.

### LORD CAVE'S COMMISSION.

The Medical Correspondent of the *Times* is responsible for the statement that the report of Lord Cave's Commission on Hospitals is expected to be issued next week. It is forecasted that the Committee will recognise that two distinct problems exist. The first concerns such institutions as the London Hospital, and it is possible that it will be recommended to the Government to make immediate grants-in-aid, free of conditions, in such instances. The second and less immediate problem concerns the present financial stringency, and it is suggested that as a temporary measure County and County Borough Associations shall be formed for the purpose of co-ordinating hospital work in each area, and also for the purpose of receiving funds. These Associations will be represented on a larger National Association, with which the Government may deal directly. This central body would distribute grants not entirely free of conditions as regards a standard of efficiency and co-operation of smaller hospitals with larger institutions in the same district. In London a central organisation, probably one of those existing, will, it is supposed, be recommended for the same work. This forecast is necessarily vague, but it contains the elements of a scheme which, it is generally supposed, will be put forward.

### THE INTERNATIONAL TUBERCULOSIS CONFERENCE.

The next of these Conferences will be held at the Institution of Civil Engineers, Westminster, from July 26 to 28 inclusive. The National Association, representing Great Britain and Ireland and the Council of the International Union against Tuberculosis, has decided to merge its Annual Conference into the International meeting, and will act as host to the International Union. The purpose of this body is to achieve an effective combination of the nations of the world against tuberculous disease, and the coming gathering gives first-class opportunity for an interchange of experienced opinion. Besides members, the Conference will be open to official delegates from countries within the League of Nations and from the U.S.A., and to representatives from all authorities concerned in this urgently important subject. Sir Alfred Mond will speak on behalf of the Ministry.

### £4,000 FOR X-RAY RESEARCH.

The *x*-ray department of the Manchester Royal Infirmary has received from an anonymous donor a gift of £4,000 in aid of research work on "Intensive X-ray Therapy." An interesting point in the conditions is that a portion of the money is to be spent on the expenses of a scholar who is to proceed to the various Continental centres, such as Erlangen, where cancer is being systematically treated by *x*-ray methods. Although complete success cannot yet be in any sense claimed for this treatment, still there is no doubt that the limit of its possibilities are still not reached. The gift has been gratefully accepted, and arrangements are being made for installation of the necessary apparatus at Manchester, while Dr. Anderson has been appointed to the Research Scholarship.

### £10,000 A YEAR FOR THE MIDDLESEX.

The Earl of Athlone, presiding at a meeting of the governors of the Middlesex Hospital, made a welcome announcement with regard to the organisation of patients' payments. He stated that receipts for the first three months show contributions at the rate of over £10,000 a year, and larger receipts are to be expected when the scheme has been longer in operation. It is necessary, he urged, that what must be got into people's heads is the fundamental truth that the benefits of hospitals are not confined to those treated within their walls, but that through research work and teaching every class is a debtor to a very tangible extent. This is a point which must be brought out very clearly in any collective scheme of publicity

### LIABILITY TO INDUSTRIAL DISEASE.

Lecturing at the Royal Society of Arts on May 30, Sir Kenneth Goadly gave some valuable hints to public authorities and trade unions. They would be well advised to institute methods by which a workman's liability to industrial disease could be determined before he actually entered dangerous trades. Our recent knowledge of the effects of a variety of deleterious substances is considerable, but we do not therefrom derive a complete insight to the personal factors in industrial disease. To the lecturer's initial suggestions that we should determine in every man the presence or absence of the normal number of white blood cells, and measure his blood pressure, one might add a large number of clinical tests. The main point is, however, that occupational selection should be made. Persons might well be assigned to that class of work for which they are most naturally fitted. Some degree of improved contentment and increased output would undoubtedly result.

### THE PRINCE AND THE MINERS' HOSPITAL.

One of the most interesting events of the Prince of Wales's Cornish tour was his visit to the Miners' and Women's Hospital, Redruth, where, in the evening of a long day, he opened the new wing, provided largely through the generosity of Sir Edward Nicholl. Major Bain received the Prince, to whom Dr. F. Hitchens and the matron, Miss M. S. Freeman, were presented. After the matron had handed him a key, and he had written his name in the visitors' book, he formally declared the new wing open, and then received a deputation of five miners from one of the mines which had been lately closed. These men, explained Mr. James Wickett, on behalf of their fellows had agreed to give up £200 a month in wages in order to keep the mine going. It is practically twenty years since the West Cornwall Miners' Hospital and the West Cornwall Women's Hospital amalgamated. The institution, which has wards for paying patients, possesses about fifty beds.

### GIFT OF A PAGE IN "PUNCH."

The heavy expenditure, which is at times out of all proportion to the results accruing therefrom, entailed at the present time by even a moderate publicity campaign, would not be such a terror to hospital secretaries if more firms were to follow the generous example of Messrs. Haig & Haig, of Southwark Street, who have defrayed the cost of a full-page appeal in *Punch*, on behalf of the Royal Waterloo Hospital. Mr. Pimm, the secretary of the hospital, informs us that the funds of the institution have already benefited to the extent of over £200. It may be confidently anticipated that this amount will be considerably augmented, as Messrs. Haig & Haig have promised the first twenty-five donors of £20 or upwards a case of their whiskey. There are, doubtless, many other firms in London who would be prepared to assist hospitals in a similar practical way if approached.

### INSURED PATIENTS AND HOSPITALS.

At a Glasgow conference of the Independent Order of Oddfellows it was decided to ask the Health Ministry to appoint a committee of all interested, including insured persons, with a view to securing a more equitable distribution of payments available for medical benefit as between panel doctor and hospitals. Lord Knutsford has had correspondence with the Mayor of Llanelly on the question of the disposal of surplus funds. The Mayor, who is a prominent Oddfellow, takes the view that the societies are not free in the matter, and are tied down by the requirements of the Audit Department, which is quite a separate Government department from the Ministry of Health. At present, according to the view of Mr. Alderman Roberts, a society would have to show that it got a pound's worth for every pound it gave to a hospital. This position coincides with a recent ruling of the Scottish Board of Health, which was "not prepared to give provisional sanction to the heads of any scheme unless they are assured that the proposals came within the terms of any of the scheduled benefits, and are likely to provide a reasonable service to members requiring the benefit." Arguably legislation is necessary before the wishes of Lord Cave's Committee can be acted upon.

### A PAGEANT IN EDINBURGH.

While the London hospitals are considering the matter of a pageant, the Royal Infirmary at Edinburgh has organised one which has met with unqualified success, and has proved a picturesque and striking spectacle. This is the second occasion upon which a pageant has been employed to bring the claims of this great Scottish hospital before the eyes of the public. From the description given it is gathered that the pageant itself had very little to do with hospitals, but represented the trades and crafts of the large number of people who took part. There was, however, one tableau depicting "Charity," which gave a hospital note to the occasion, and ambulances also played a part in this respect. Although we expect the pageant in London will not be of so mixed a character, there is a great deal to be learned from the Edinburgh venture, in which the collection of contributions from the public was seemingly very well organised.

### PAYING PATIENTS AT CAMBERWELL INFIRMARY.

Dr. Masterman, superintendent of Camberwell Infirmary, in his annual report, deals with the case of Camberwell residents needing hospital treatment who are either not entitled to come into the infirmary under the Poor-law or who do not wish to do so. He recommends that when the needs of the poor have been fully met such persons should be admitted as private paying patients, making their own arrangements for admission with the medical superintendent, and paying direct to the infirmary. He suggests that three guineas per week should be the minimum payment, and that for this such patients should be entitled to ordinary diet, nursing, and medical attendance; special diet, stimulants, or unusual medicines or apparatus required to be paid for as extras, together with any outside consultant advice desired. He deems it essential to the success of such a scheme that "such patients should have nothing to do with the ordinary routine of 'admission orders,' relieving officers, etc." On two occasions the Infirmary Committee have considered this matter, and eventually came to the conclusion that they were unanimously in full accord with the proposals. The Board have ratified the opinion of the Committee, and it now remains to be seen whether the sanction of the Ministry of Health can be obtained to it.

### HOSPITAL SECRETARY JOINS THE BOARD.

Mr. D. D. Kirkaldy-Willis has resigned the Secretaryship of the West End Hospital for Nervous Diseases, Welbeck Street, and has been invited to join the Committee of Management.

### COST OF MAINTENANCE AND GIFTS IN KIND.

Notwithstanding the official figure and the admitted decline in the price of certain foods, the cost of provisions during the March quarter at Addenbrooke's Hospital, Cambridge, showed a small increase. This seems to be due in part to the diminution of gifts in kind which followed a bad fruit harvest and necessitated further purchases, and in part to a slight increase in the numbers of patients and staff. Mr. W. H. Head, the secretary and superintendent, is able to report certain increases, the most remarkable item being 23,000 eggs from the latest collections in the county. Addenbrooke's, as we have often pointed out, has been conspicuous for its success in organising gifts in kind, and the value of these is shown from a new angle when the accounts show an increase in the cost of provisions because circumstances have interfered with the usual number of gifts received.

